# Choose the appropriate implantation position of the Femoral Neck System in the femoral neck: a finite-element analysis

**DOI:** 10.1007/s00068-023-02253-5

**Published:** 2023-03-25

**Authors:** Ximu Kuang, Guojian Jian, Desheng Xie, Xiaolin Chen, Haoyuan Liu

**Affiliations:** Department of Orthopedics, Chenggong Hospital of Xiamen University, School of Medicine, Xiamen University, Xiamen, China

**Keywords:** Femoral neck fracture, Femoral Neck System, Finite-element analysis, Young adults, Implantation position

## Abstract

**Purpose:**

There is no specific literature on the best implantation position of the Femoral Neck System (FNS) for treating Pauwels type III femoral neck fracture in young adults.

**Methods:**

Use finite-element analysis to compare the mechanical properties of implantation positions: FNS in the central position, FNS in the low position, and FNS in the low position combined with cannulated screw (CS). The CT data of the femur were imported into the mimics20.0 to obtain the three-dimensional model of the femur; imported into geomagic2017 and SolidWorks 2017 for optimizations; models of FNS and CS are built on the basis of the device manuals. Grouping is as follows: FNS group, FNS-LOW group, and FNS-CS group. Assemble and import them into abaques6.14 for load application. The displacement distribution and von Mises Stress value of them were compared.

**Results:**

On femoral stability and stress distribution, the FNS-CS group performs best, followed by the FNS-LOW group, and finally FNS group. The FNS-LOW group has an improvement over the FNS group but not by much.

**Conclusion:**

In operations, when the implantation position of the central guide wire is not at the center of the femoral neck but slightly lower, it is recommended not to adjust the wire repeatedly in pursuit of the center position; for femoral neck fractures that are extremely unstable at the fracture end or require revision, the insertion strategy of FNS in the low position combined with CS can be adopted to obtain better fixation effects.

## Introduction

There are about 1.6 million hip fractures annually; these data increases by 25% every 10 years [1]. Hip fractures have become one of the significant financial burdens of the healthcare system. Among them, femoral neck fracture is one of the common traumas in the clinic, and its incidence is increasing with the aging of the population and urban modernization. It is characterized by poor recovery and quite a few complications, once known as “unresolved fractures” [2].

With the development of orthopedic technology and materials science, we have gradually made a strategy for treating elderly femoral neck fractures with simple internal fixation, hemiarthroplasty, or total hip arthroplasty. When faced with femoral neck fractures in young people [3], we advocate using internal fixation for hip-preserving treatment strategies. Internal fixation devices such as three cannulated screws (CS), dynamic hip screws (DHS), and medial anatomical buttress plates aim to achieve strong fixation. CS and DHS have been the first choice for treating displaced or non-displaced femoral neck fractures in young adults [4]. They have their advantages in anti-rotation, promoting fracture healing, etc. However, the incidence of fixation failure, nonunion, and necrosis of the femoral head after the operation remains high.

Combined with CS’s less invasiveness and the DHS’s stability, Depuy Synthes medical device company has developed a brand new product, the Femoral Neck System (FNS). Its design strategy is to reduce the incidence of complications and secondary surgery by increasing stability, reducing invasiveness, and reducing the risk of lateral protrusion. Currently, the operation of femoral neck fractures treated with FNS has been performed clinically, and specific therapeutic effects have been observed.

During the actual surgical operation, there are also some doubts, such as the implantation position of the bolt. Reading the Femoral Neck System Surgical Technique and the relevant literature on surgery in the database, we can find that it is currently recommended to implant the internal fixation device in the center of the femoral neck so that an excellent bone-holding force and mechanical stability can be obtained; the device is generally not recommended to be placed in a position above the center of the femoral neck, which may cause the device to cut out the femoral neck. Due to human factors, we cannot achieve the above standard position. However, no literature currently tells us the appropriate location other than a central one. Choose the appropriate implantation position of the FNS in the femoral neck is what we investigated.

The finite-element analysis uses mathematical methods to simulate the actual physical system [5]. In this experiment, finite-element analysis will establish a femoral neck fracture model. Then, the mechanical characteristics of the implantation method of FNS in the central position, low position, and FNS in the low position combined with CS will be compared.

## Methods

### Three-dimensional (3D) modeling of the femoral neck fracture

#### 3D modeling of the femur

One subject (the author himself), male, 26 years old, height 170 cm, weight 70 kg, was selected; use X-rays to examine bilateral femurs to rule out fractures, deformities, and tumors; use 32-slice spiral CT to scan from the distal end of the femur to the femoral head from bottom to top. The CT image layer thickness is 1.0 mm, and the image data were imported into Mimics20.0 and Geomagic 2017 software for 3D reconstruction and optimization, respectively. Finally, a femur model with a diaphyseal angle of 130.96° and an anteversion angle of 13.34° was obtained.

#### 3D modeling of the Pauwels type III femoral neck fracture

**Fig. 1:**
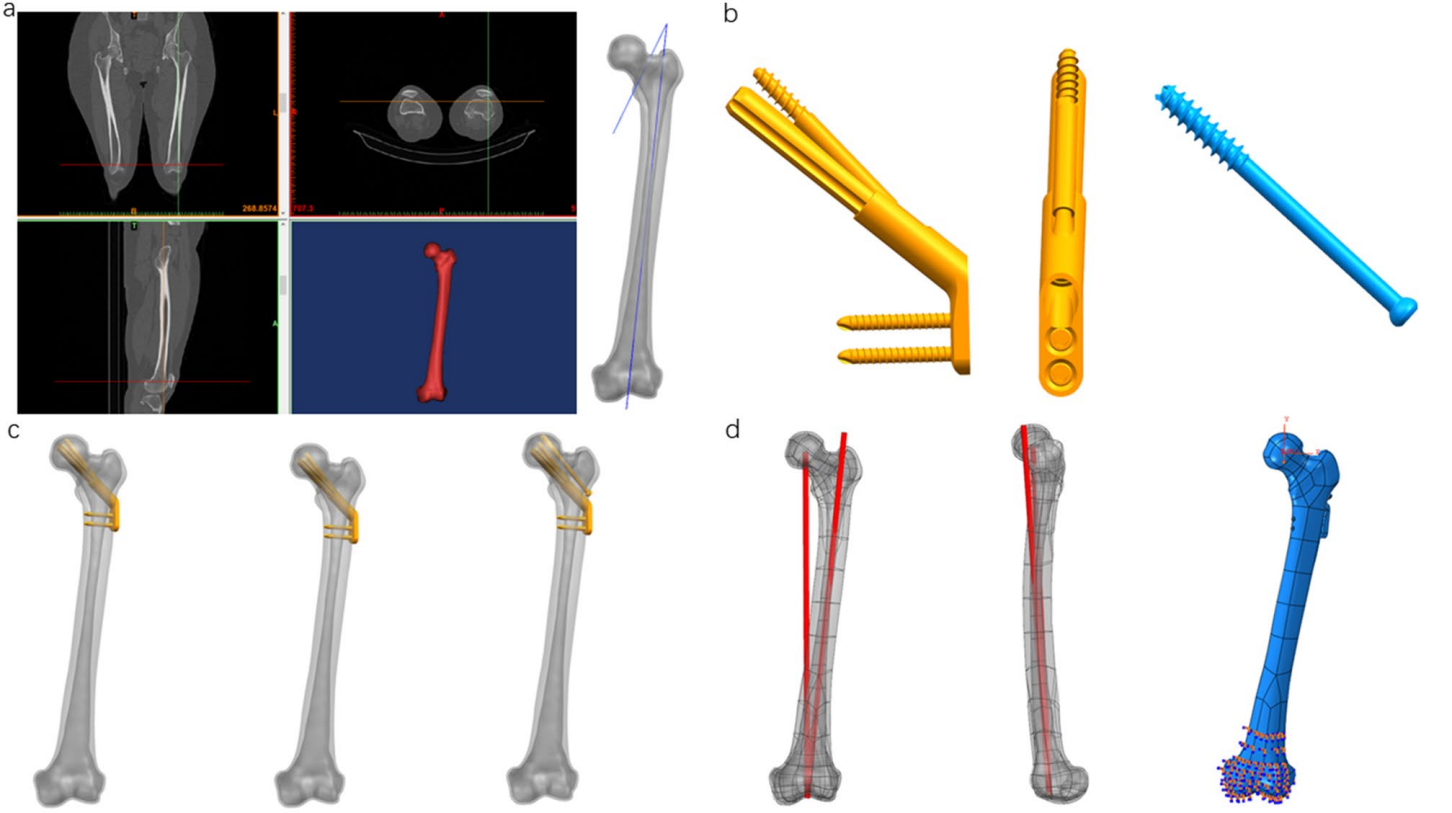
Establishment of the model and force analysis. **a** CT scan process and establishment of the model of femoral neck fracture (Pauwels III). **b** The establishment of the model of internal fixations. **c** The assembly of the internal fixation model and femoral neck fracture model. **d** Lower extremity line of force and the direction where the loads are applied

Pauwels classification divides femoral neck fractures into three types according to the angle between the fracture line and the horizontal line: < 30° is type I, 30 to 50° is type II, and > 50° is type III [6]. Femoral neck fractures in young adults are mostly Pauwels type III fractures with high shear force type, so this model dealt with Pauwels 70° (Fig. 1a).

## 3D modeling of internal fixation model

### 3D modeling referring to the internal fixation device manual

FNS: Referring to the Femoral Neck System Surgical Technique provided by Depuy Synthes, the FNS model was constructed in SolidWorks 2017 software.

CS: Referring to the implants in the 6.5 mm and 7.3 mm cannulated screw system provided by Depuy Synthes Medical Company, the CS model was constructed (Table 1).

**Table 1:** Introduction of implant materials

Contents/internal fixation device	FNS (Femoral Neck System)	CS (cannulated Screw)
Manufacturer	Synthes	Synthes
Medical device registration number	20163131218	20193130357
Material	Titanium alloy (Ti6A17Nb)	Titanium alloy (Ti6A17Nb)

Subsequent research will set the screw and the bone in a binding contract relationship. The thread has little effect on the experimental results, so the threaded part is removed. The thread is provided with a smooth surface, the diameter of which corresponds to the thread diameter (Fig. 1b).

### Assembly of internal fixation and the femoral neck fracture model

Grouping situation: FNS group: the FNS internal fixation device was placed in the center of the femoral neck.

FNS-LOW group: the FNS internal fixation device was placed in the low of the femoral neck.

FNS-CS group: the FNS internal fixation device is placed in the low of the femoral neck. Insert the cannulated screw in a limited position between the FNS anti-rotation screw and the cortex above the femoral neck, the distal end of which is less than 5 mm from the cortex of the femoral head.

The assembled model has been imported into Abaqus 6.14 software for automatic mesh division processing. The number of nodes and elements of the finite-element model are shown in Table 2 (Fig. 1c).

**Table 2:** Number of nodes and elements of two finite-element models

Finite-element model	Number of nodes	Number of elements
FNS	99,911	58,124
Screw	12,667	7542
Femur	189,950	122,813

## Material parameters

Due to the irregular shape of the proximal femur and the uneven bone quality, the material of the femur model is simplified. All models are assumed to be continuous, isotropic, homogeneous linear elastic materials [7, 8] (Table 3).

**Table 3:** Material parameters of the model

Material	Elastic modulus (MPa)	Poisson’s ratio
Femoral cortical bone	16,800	0.3
Femoral cancellous bone	840	0.2
Titanium alloy (Ti6A17Nb)	110,000	0.3

## Boundary conditions

In order to simulate the stress of the femoral neck under a standing posture, the distal end of the femur is wholly fixed: all nodes are constrained to 0 degrees of freedom. Assuming a complete fracture of the femoral neck without displacement or simulating anatomical reduction, the fracture contact surface was set to a friction relationship with a friction coefficient of 0.2 [9]. Set the internal fixation device in binding contact with the femoral neck.

## Loading force settings

Referring to the existing literature, in order to simulate the stress of standing on one foot during normal walking, the direction in which the load is applied is 10° outward inclined in the coronal plane and 9° backward in the sagittal plane [10]. A load of 2100 N, approximately equal to three times the body weight, is applied vertically downward at the center of the femoral head (Fig. 1d).

## Evaluation criteria

The indicators recorded and analyzed in this experiment include (1) displacement distribution and peak value of the femur; (2) VMS distribution and peak value of the femur; (3) displacement distribution and peak value of the three internal fixations; (4) VMS distribution and peak value of the three internal fixations; (5) displacement, compressive stress, and rotation angle between the fracture fragments.

## Results

### Displacement distribution and peak value of femur

The displacement peak values of the femurs in the three groups of models were all located at the top of the femoral head and gradually decreased from the femoral head to the distal end of the femur. The displacement peak value of the femoral head in the FNS-CS group was reduced by 5.1% compared with the FNS group (Table 4).

**Table 4:** Specific data for the experiment

Contents/groups	FNS	FNS-LOW		FNS-CCS	
			Variation		Variation
The maximum displacement of the femur (mm)	4.88	4.84	0.82%	4.63	5.1%
The maximum displacement of the internal fixation(mm)	4.77	4.58	4.00%	4.51	5.45%
The maximum Von Mises stress (VMS) values of the femur (MPa)	48.38	45.56	7.70%	34.40	30.33%
The maximum Von Mises stress (VMS) values of the internal fixation (MPa)	655.2	628.8	4.02%	496.9	24.16%
The maximum displacement of fracture surface(mm)	0.22	0.161	26.80%	0.136	38.20%
The maximum compressive stress of fracture surface (MPa)	28.75	41.05	** − 42.80%**	12.73	55.72%
The maximum rotation angle (°)	2.23	2.16	3.10%	1.85	17.00%

### VMS distribution and peak value of the femur

**Fig. 2:**
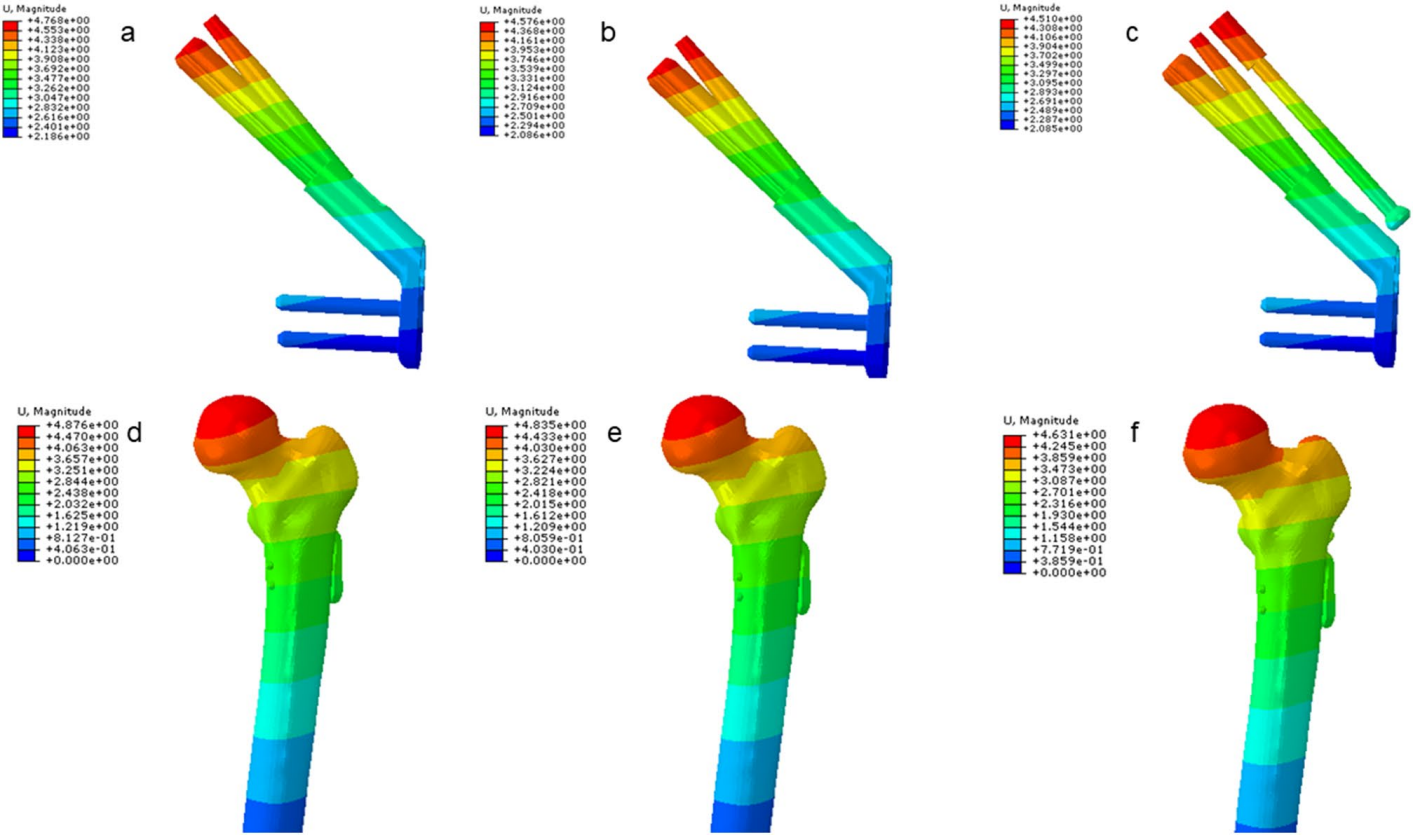
The displacement distribution of internal fixations and femur

The VMS value of the femur in the three groups of models was mainly concentrated below the femoral neck and gradually decreased from proximal to distal, with the fracture end as the center. The VMS peak value in the FNS-LOW group was reduced by 7.7% compared with the FNS group, and the FNS-CS group was decreased by 30.33% compared with the FNS group (Table 4 and Fig. 2).

### Displacement distribution and peak value of the three internal fixations

The internal fixation displacement in the three model groups was mainly concentrated at the top of the internal fixation device and gradually decreased from near to far. The displacement peak value of internal fixation in the FNS-LOW group was reduced by 4.00% compared with the FNS group, and the FNS-CS was decreased by 5.45% compared with the FNS group (Table 4).

### VMS distribution and peak value of the three internal fixations

**Fig. 3:**
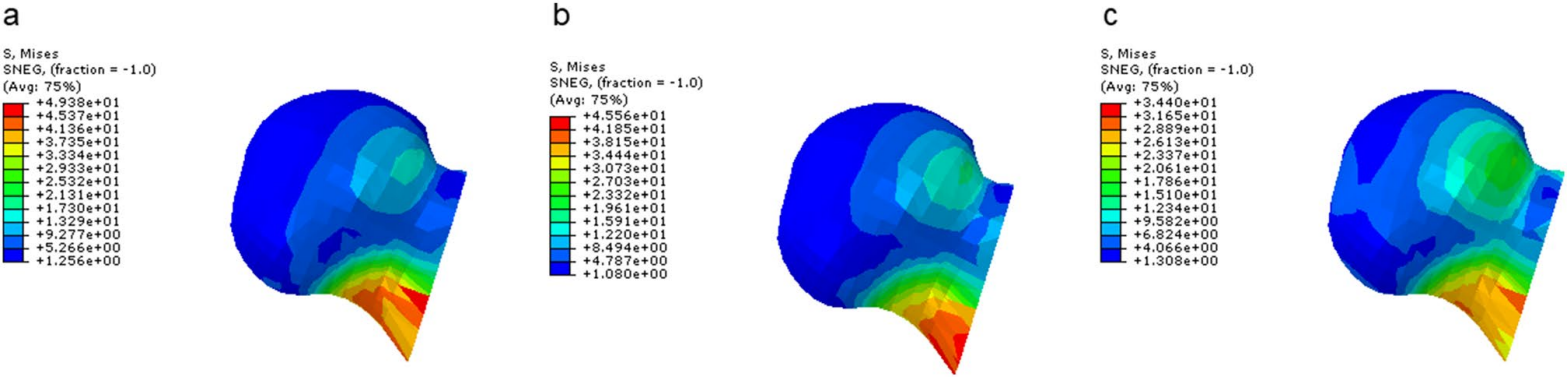
The distribution of Von Mises stress (VMS) values of the femur

**Fig. 4:**
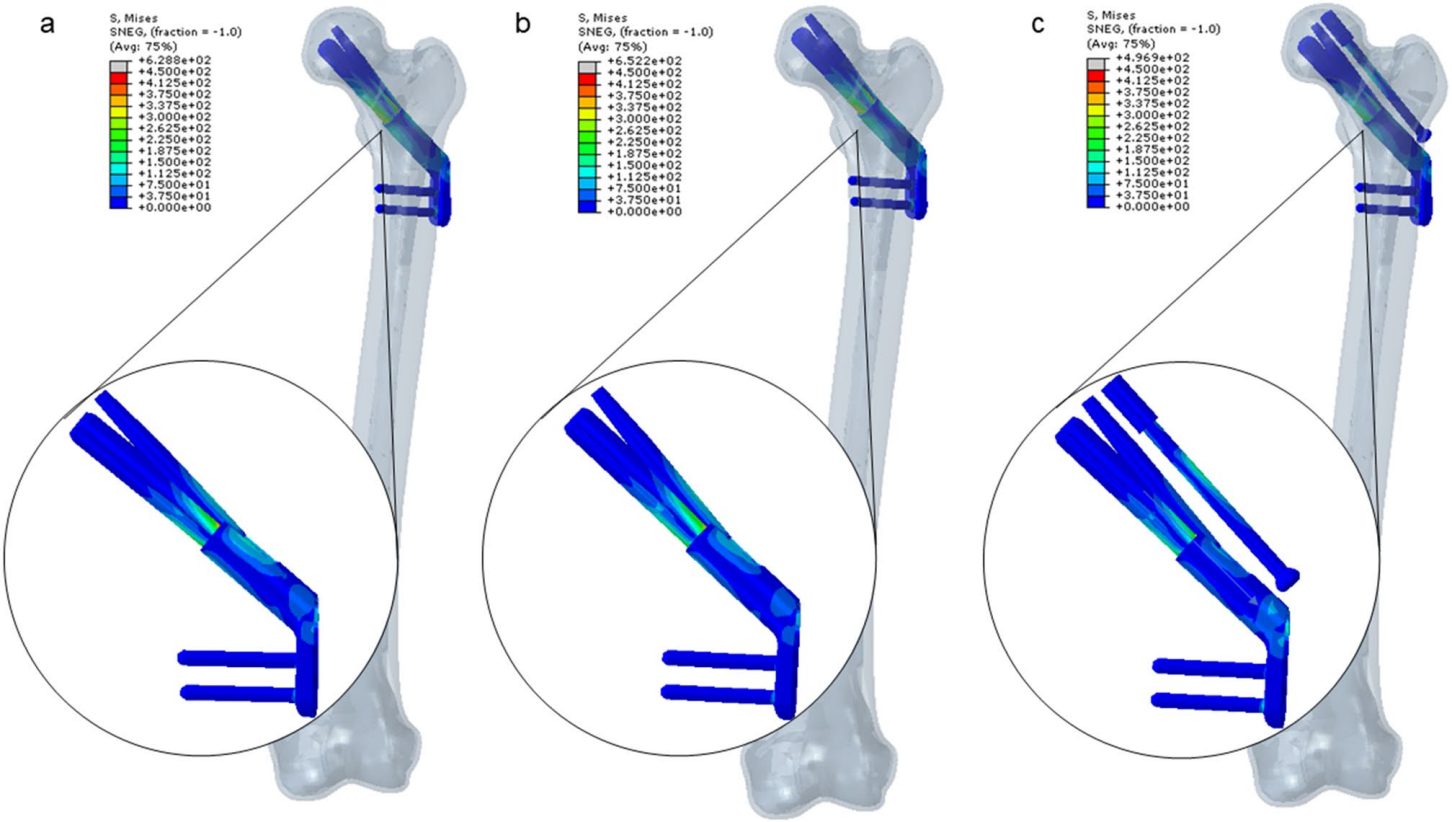
The distribution of Von Mises stress (VMS) values of internal fixations

The VMS value of internal fixation in the three groups of models was concentrated in the position of the screw at the fracture end and the transition of the plate-sleeve. The VMS peak value of the plate in the FNS-LOW group is reduced by 14.40% compared with the FNS group; compared with the FNS group, the VMS peak value of the bolt is reduced by 21.16%, the anti-rotation screws are reduced by 44.15%, and the plate is reduced by 50.24% (Table 5 and Figs. 3 and 4).

**Table 5:** The maximum Von Mises stress (VMS) values of elements

Contents/groups	FNS	FNS-LOW		FNS-CCS	
			Variation		Variation
Bolt (MPa)	652.2	628.8	4%	496.9	21.16%
Anti-rotation screw (MPa)	314.4	312.2	0.07%	175.6	44.15%
Plate (MPa)	459.6	393.4	14.40%	228.7	50.24%
Locking screw (MPa)	142.0	143.5	− 1.1%	139.8	1.5%
7.3 mm CCS (MPa)	/	/	/	188.6	/

### Displacement, compressive stress, and rotation angle between the fracture fragments

**Fig. 5:**
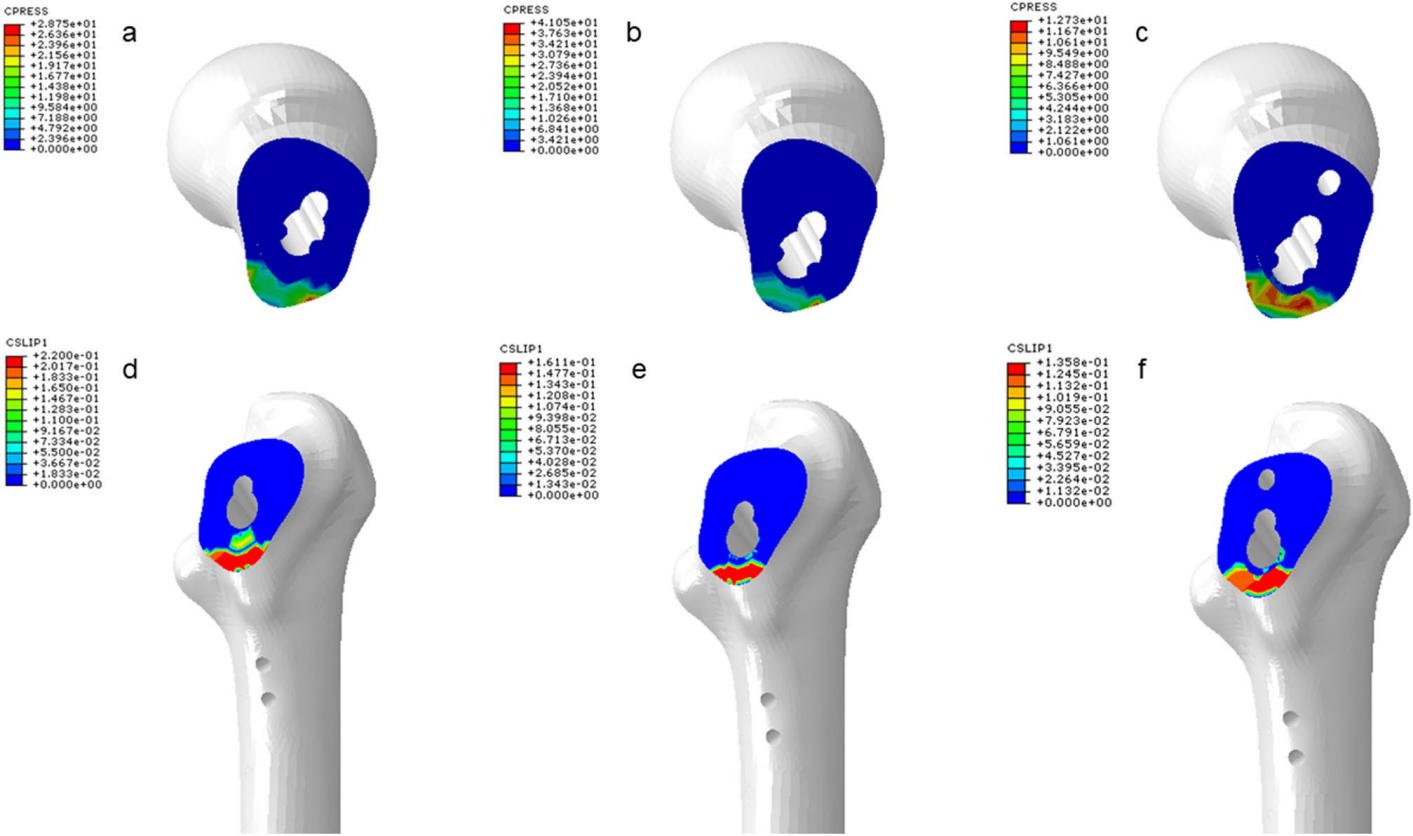
The compressive stress and displacement of the fracture surface

The displacement peak value of the FNS-LOW group was reduced by 26.8% compared with the FNS group, and the FNS-CS group decreased by 38.2% compared with the FNS group; the compressive stress peak value of the FNS-LOW group was increased by 41.80% compared with the FNS group, and the FNS-CS group was reduced by 55.72% compared with the FNS group; the maximum rotation angle in the FNS-CS group was decreased by 17% compared with the FNS group (Table 4 and Fig. 5).

## Discussion

Recently, the occurrence of femoral neck fractures in young is increasing. The muscle-pulling force leads to high shear force at the femoral neck, and the fracture fragments are displaced in the vertical direction, forming unstable fractures [11]. Femoral neck fractures in young adults often present as Pauwels type III fractures and generally require surgical treatment. CS and DHS have advantages in anti-rotation and maintaining stability, but postoperative complications are still high. Based on the available literature, the failure rate of CS is as high as 13% [12]; 5.3% [13] of patients with lateral thigh pain caused by screw withdrawal make the overall secondary operation rate as high as 33% [14]; the fixation failure rate of DHS is reduced to 5% compared to CS, but there is a 10% postoperative infection rate and an overall 22% secondary surgery rate. Preclinical experiments such as biomechanical analysis or finite element analysis conducted by Karl Stoffel et al. [7], Schopper et al. [15], etc. and many clinical experiments [16–18] have proved the excellent mechanical properties and good treatment results of FNS. The correction guide included in the FNS device is designed to assist us in accurately inserting the guide wire, but in practice, deviations may occur due to human-operated implantation. Computer-aided technology or 3D printing guide technology can solve these problems to a certain extent, but in ordinary hospitals, the equipment is relatively insufficient and relies more on guidelines and experience. The current doubt is: if the bolt is placed at a position lower than the center of the femoral neck, that is, near the more inferior cortex of the femoral neck. What is the stability of the femur under this fixation position? There is currently no relevant literature to describe it. The practical significance of this question is: during the operation, if the central guide wire has been implanted in the above position due to artificial deviation for a reduction of frequency of insertion of the central guide wire and control the operation time, is it necessary to take it out and adjust it? If no adjustment is required, is it still possible to implant an additional cannulated screw over the anti-rotation screw to combat the high shear forces of femoral neck fractures in young adults when there is enough space in the femoral neck? In order to cope with the high shear force, whether the strategy of FNS combined with CS can be routinely chosen when we deal with the femoral neck fracture in young people; in the case of the first internal fixation failure, whether FNS combined with CS and FNS in the position of low can be used as a suitable revision plan, there is currently no relevant experiments and literature illustrating about doubts above. Therefore, what is the difference in the mechanical properties among the three implantation positions of “FNS in the central position”, “FNS in the low position”, and “FNS in the low position combined with CS”, determines our surgical process to a certain extent, which is also the focus of this experiment.

Analyzing the experimental data, in the case of simulating human standing on one foot, the VMS peak value of the femur internal fixation shows that the three fixation positions can effectively bear most of the stress of the lower limbs and provide conditions for fracture healing. The stress of internal fixation in the three groups of models is mainly concentrated in the middle part of the screw close to the fracture line, followed by the transition of the plate-sleeve, which is consistent with that reported in the literature by Li et al. [16] and Fan et al. [19]. This also shows that the idea that adding a CS above the FNS internal fixation device in this experiment to resist better the shear force at the fractured end of the femoral neck is correct.

From the experimental data, we can find that both the FNS-LOW group and the FNS-CS group reduced the stress of the femur to a certain extent compared with the FNS group; among them, the method of FNS in the low position combined CS significantly (30.33%) reduced the VMS value of the femur. The pressure values on the overall structure of the internal fixation or each element in the FNS-LOW group and the FNS-CS group were lower than those in the FNS group to a certain extent (except that the locking screws in the FNS-LOW group increased by 1.1% compared with the FNS group). Referring to the internal fixation stress distribution map (Fig. 4), we can see that the lower implantation position of the device and the added CS make the stress distribution on the internal fixation device more reasonable, thereby reducing the risk of damage to the device.

Regarding displacement of the femur, the FNS-LOW group and the FNS-CS group performed better than the FNS group. Among them, there was little difference (0.82%) in the displacement of the femur between the FNS group and the FNS-LOW group. In the two parts of the fracture end's maximum displacement and maximum rotation angle, the FNS-LOW group and FNS-CS group had significant changes compared with the FNS group. The compressive stress peak value of the FNS-LOW group was increased compared with the FNS group, and the FNS-CS group was reduced compared with the FNS group. A certain degree of compressive stress in the fracture end facilitates fracture healing. Therefore, we can indicate that the implantation of FNS in the low position can promote fracture healing compared to the implantation of FNS in the central position. As for the data of the FNS-CS group, we speculate that it may be the effect of more internal fixation dispersing the fracture end stress.

“Anatomical reduction,” “Fracture fixation providing absolute or relative stability,” “Preservation of the blood supply to soft tissues and bone,” and “Early and safe mobilization and rehabilitation” are the four principles for the treatment of fractures [20]. Mechanical stability is the initial factor of fracture healing [21]. In order to achieve strong fixation, on one hand, we choose an internal fixation device with excellent mechanical properties. On the other hand, we will pursue a better fixation position for the internal fixation device, such as the inverted triangle fixation method of CS, and the blade in PFNA close to the lower part of the femoral neck. However, obtaining the ideal position is difficult in the actual operation process, and it is necessary to adjust the guide wires several times. The research of MEI JIONG et al. [22] tells us that the adjustment times of the guide wires should not exceed 14 times; otherwise, there will be a risk of reduced bone strength and insufficient screw holding force. Repeated insertion of the guide wire may damage the blood supply of the femoral head, thereby increasing the risk of femoral head necrosis [23]. Not only that, repeatedly adjusting means that the patient is exposed to the X-ray machine for more time [24]. This increases the operation time and the risk of contamination of the surgical area, which may lead to postoperative infection. In the surgical treatment of patients with femoral neck fractures, the overall return of surgery should be considered: shorter operation time, less bone and vascular damage, mechanically excellent internal fixation, and its appropriate insertion location; combining the three can provide better conditions for fracture healing.

### Limitation

There are also some limitations in this experiment. This finite-element analysis has made certain simplifications in modeling and load application, such as the muscles around the femur, the cortical bone and cancellous bone, the force under dynamic walking, etc., and does not fully simulate the actual pressure and natural structure of bones. This study aims to compare the overall change trend of stability. It focuses on studying whether this way is better rather than whether this internal fixation is reliable, which has already been discussed, so its data still have specific reference significance; all questions above will be the direction of follow-up research. Besides, biomechanical experiments and clinical case data need to verify these results.

## Conclusion

In applying FNS in treating femoral neck fractures in young adults, we used the finite element analysis method to compare three internal fixation implantation positions: FNS in the central position, low position, and FNS in the low position combined with CS. The results showed that, in terms of femoral stability and internal fixation stress distribution, the implantation method of FNS in the low position combined with CS is the best, followed by FNS in the low position and FNS in the central position. The implantation method of FNS in the low position has an improvement over that in the central position but not by much. In actual clinical operations, when the implantation position of the central guide wire is not at the center of the femoral neck but slightly lower, on the premise of ensuring that the subsequent bolt insertion will not cut out the femoral neck, it is recommended not to adjust the wire repeatedly in pursuit of the center position; for femoral neck fractures that are extremely unstable at the fracture end or require revision, the insertion strategy of FNS in the low position combined with CS can be adopted to obtain better fixation effect.

## Data Availability

The datasets supporting the conclusions of this article are included within the article.
